# Caspase-4 in glioma indicates deterioration and unfavorable prognosis by affecting tumor cell proliferation and immune cell recruitment

**DOI:** 10.1038/s41598-024-65018-z

**Published:** 2024-07-29

**Authors:** Longjiang Di, Mengyan Li, Xianli Lei, Wenting Xie, Guoqiang Liu, Yongqing Wang, Wenjing Zhang, Wei-Guo Zhu

**Affiliations:** 1 School of Basic Medical Sciences, Southern Medical University, Guangzhou, 510515 China; 2 Guangdong Key Laboratory of Genomic Instability and Human Disease Prevention, Department of Biochemistry and Molecular Biology, School of Medicine, Shenzhen University, Shenzhen, 518055 China; 3 Department of Biochemistry and Biophysics, School of Basic Medical Sciences, Faculty of Medicine, Peking University, Beijing, 100191 China; 4 Department of Clinical Laboratory, Second Affiliated Hospital of Harbin Medical University, Harbin, 150086 Heilongjiang China; 5 Division of Rheumatology and Immunology, University of Toledo Medical Center, Toledo, OH 43614 USA; 6 College of Basic Medical Sciences, Wan Nan Medical College, Wuhu, 241006 China; 7 International Cancer Center, School of Medicine, Shenzhen University, Shenzhen, 518055 China

**Keywords:** Glioma, CASP4, Immunotherapy, Chemotherapy, Pyroptosis, Cancer, Cancer screening, Cancer therapy, CNS cancer, Tumour immunology

## Abstract

Gliomas are the most common malignant tumors of the central nervous system, accounting for approximately 80% of all malignant brain tumors. Accumulating evidence suggest that pyroptosis plays an essential role in the progression of cancer. Unfortunately, the effect of the pyroptosis-related factor caspase-4 (CASP4) on immunotherapy and drug therapy for tumors has not been comprehensively investigated. In this study, we systematically screened six hub genes by pooling differential pyroptosis-related genes in The Cancer Genome Atlas (TCGA) glioma data and the degree of centrality of index-related genes in the protein–protein interaction network. We performed functional and pathway enrichment analyses of the six hub genes to explore their biological functions and potential molecular mechanisms. We then investigated the importance of CASP4 using Kaplan–Meier survival analysis of glioma patients. TCGA and the Chinese Glioma Genome Atlas (CGGA) databases showed that reduced CASP4 expression leads to the potent clinical deterioration of glioma patients. Computational analysis of the effect of CASP4 on the infiltration level and recruitment of glioma immune cells revealed that CASP4 expression was closely associated with a series of tumor-suppressive immune checkpoint molecules, chemokines, and chemokine receptors. We also found that aberrant CASP4 expression correlated with chemotherapeutic drug sensitivity. Finally, analysis at the cellular and tissue levels indicated an increase in CASP4 expression in glioma, and that CASP4 inhibition significantly inhibited the proliferation of glioma cells. Thus, CASP4 is implicated as a new prognostic biomarker for gliomas with the potential to further guide immunotherapy and chemotherapy strategies for glioma patients.

## Introduction

Gliomas are the most common heterogeneous tumors of the central nervous system (CNS), accounting for 81% of CNS malignancies, and are responsible for most deaths from primary brain tumors^1–3^. Traditional treatments for gliomas, such as appropriate chemotherapy and immunotherapy agents, are considered the most effective clinical strategies^4,5^, However, these approaches have limited anticancer effects in glioma patients, contributing to their poor prognosis^6,7^. Since the treatment of gliomas continues to be a challenge for clinicians around the world, it is important to understand the mechanisms involved in glioma development and progression. In addition, the identification of new markers that synergize with conventional treatment approaches for clinical gliomas represents a promising new therapeutic strategy.

Pyroptosis is a recently discovered form of cell death that occurs through the formation of inflammasomes and activation of the caspase protein cascade. These processes are accompanied by the cleavage of the gasdermin family and release of cell contents including pro-inflammatory factors (e.g., IL1β and IL18) via pores formed in the cell membrane, thereby promoting inflammatory and immune responses^8^. In 2020, Erkes et al. found that BRAF and MEK inhibitors promote T cell infiltration in the immune microenvironment of melanomas through pyroptosis, thereby increasing the sensitivity of the tissue to drug activity and this approach has been widely used in clinical treatment^9^. An increasing number of studies have shown that levels of cellular pyroptosis can be used to predict cancer prognosis, and indicate the occurrence of immune cell infiltration. These studies demonstrate the importance of cellular pyroptosis-related genes in screening for relevant biological targets in the context of radiotherapy, chemotherapy, and immunotherapy for cancers.

Caspase-4 is a member of the caspase family of proteins. The caspase (aspartate-specific cysteine protease) family contains at least 12 members (including CASP1, CASP3, and CASP4) that have a significant impact on cell death, inflammatory responses, the immune microenvironment, and tumor suppression^10^. Caspase-4 (CASP4) has been shown to amplify inflammation and generate cellular pyroptosis in a number of diseases^11,12^. Furthermore, an association between CASP4 levels and outcomes after tumor treatment has been identified. In esophageal squamous cell carcinoma, loss of CASP4 expression has been associated with poor prognosis^13^. However, in epithelial tumors and pancreatic cancer, CASP4 has been shown to play an integral role in promoting tumor cell migration, cell–matrix adhesion and tissue invasion^14^. The specificity of the correlation between CASP4 expression and outcomes in different tumors indicates the potential for synergistic effects with radiotherapy, chemotherapy and immunotherapy in gliomas.

Changes in the tumor microenvironment (TME) are the main cause of tumor progression, and agents that modulate immune cells infiltrating the TME have become critical components of tumor immunotherapy^15^. The glioblastoma microenvironment contains a large number of innate immune cells (e.g., NK cells, and macrophages)^16^, which play important roles in the progression and treatment-resistance of glioma^17^. The TME of glioma has a complex dynamic pattern of communication with tumor cells that is critical for tumor proliferation, migration, and immunosuppression^18^. Furthermore, the inflammatory state of the TME can influence responses to immune checkpoint therapy^19–21^. With the widespread clinical use of immune checkpoint blockade (ICB) therapy and its positive impact on treatment outcomes, it has attracted increasing attention in the treatment of glioma^22,23^.

In this study, we screened for a series of genes associated with pyroptosis in gliomas using TCGA data. Through integrated bioinformatics analysis techniques and evaluation of the prognostic significance of pivotal pyroptosis genes, we identified CASP4 as a gene that is critical to glioma pyroptosis. We then used comprehensive analysis and multiple visualization methods to explore the mechanisms by which CASP4 affects the outcomes of glioma therapy. We conducted a series of studies, including analyses of clinical survival prognosis, functional enrichment, and patient staging, to determine the role of CASP4 in gliomas. We also analyzed the correlation between CASP4 expression and glioma immune cell infiltration using The Tumor Immuno Estimation Resource (TIMER) database to explore the potential function of CASP4 in glioma chemotherapy and immunotherapy. Moreover, we experimentally validated the function of CASP4 at the glioma cell and tissue levels. Our findings provide a greater understanding of glioma and new insights into possible strategies for chemotherapy and immunotherapy of glioma.

## Materials and methods

### Data sources and processing

Data (including mRNA expression, mutations) for gliomas up to May 2023 were downloaded from TCGA (https://portal.gdc.cancer.gov/), a platform containing gene expression profiling data and clinical follow-up information for 530 patients with low-grade glioma (LGG), 167 patients with glioblastoma (GBM), and five neighboring non-tumor tissues. The 22 pyroptosis-related genes differentially expressed between tumor and normal tissues were evaluated using the “Limma” package (version 4.0.2) of R software. The 22 pyroptosis-related genes considered in our study were identified by searching literature published in the previous 3 years (Table 1). Thresholds were set based on the following parameters: cancer versus normal tissue; *P*-value, 0.05; and a *t*-test was used to calculate *P*-values, with *P* < 0.05 considered to indicate a statistically significant difference. We also used the CGGA (http://www.cgga.org.cn/) to investigate the expression pattern of CASP4 as well as its clinical and prognostic significance in glioma patients. We collected data for two glioma cohorts (LGG and GBM), including gene expression, clinical information, grade, isocitrate dehydrogenase (IDH) mutation status, 1p/19q code deletion status, and methylation status, as independent test sets. In addition, we recruited patients with gliomas in our clinic to validate our results.

**Table 1:** Details of the 22 possible pyroptosis-related genes that have been studied in tumor patients in recent years.

Symbol	Description	Author	PMID	References
*TP53*	Tumor protein P53	Tan LL, Jiang XL, et al. (2021)	33,269,748	TP53-induced glycolysis and apoptosis regulator alleviates hypoxia/ischemia-induced microglial pyroptosis and ischemic brain damage. Neural Regen Res. 2021;16(6):1037–1043
*HMGB1*	High Mobility Group Box 1	Satoh TK (2022)	35,907,655	The role of HMGB1 in inflammatory skin diseases [published online ahead of print, 2022 Jul 13]. J Dermatol Sci. 2022;S0923-1811(22)00,167–0
*CASP6*	Caspase 6	Zheng M, Karki R, et al. (2021)	34,740,613	Caspase-6 promotes activation of the caspase-11-NLRP3 inflammasome during gram-negative bacterial infections. J Biol Chem. 2021;297(6):101,379
*NLRP3*	NLR Family Pyrin Domain Containing 3			
*BAX*	BCL2 Associated X, Apoptosis Regulator	Li RY, Zheng ZY, et al. (2022)	35,525,317	Cisplatin-induced pyroptosis is mediated via the CAPN1/CAPN2-BAK/BAX-caspase-9-caspase-3-GSDME axis in esophageal cancer. Chem Biol Interact. 2022;361:109,967
*CASP3*	Caspase 3			
*CASP1*	Caspase 1	Santoni K, et al. (2022)	35,849,616	Caspase-1-driven neutrophil pyroptosis and its role in host susceptibility to Pseudomonas aeruginosa. PLoS Pathog. 2022;18(7):e1010305
*IL6*	Interleukin 6	Gou X, Xu W, et al. (2022)	35,297,653	IL-6 prevents lung macrophage death and lung inflammation injury by inhibiting GSDME- and GSDMD-mediated pyroptosis during pneumococcal pneumosepsis. Microbiol Spectr. 2022;10(2):e0204921
*GSDMD*	Gasdermin D			
*CASP4*	Caspase 4	Kobayashi T, et al. (2022)	35,778,597	Bexarotene-induced cell death in ovarian cancer cells through Caspase-4-gasdermin E mediated pyroptosis. Sci Rep. 2022;12(1):11,123
*CHMP6*	Charged Multivesicular Body Protein 6	Xing M, Li J (2022)	35,562,678	Diagnostic and prognostic values of pyroptosis-related genes for the hepatocellular carcinoma. BMC Bioinformatics. 2022;23(1):177
*GPX4*	Glutathione Peroxidase 4			
*NLRP2*	NLR Family Pyrin Domain Containing 2	Ding MR, Qu YJ, et al. (2022)	35,658,846	Pyroptosis-related prognosis model, immunocyte infiltration characterization, and competing endogenous RNA network of glioblastoma. BMC Cancer. 2022;22(1):611
*TP63*	Tumor Protein P63			
*AIM2*	Absent In Melanoma 2	Uresti-Rivera EE, et al. (2022)	35,880,661	AIM2-inflammasome role in systemic lupus erythematous and rheumatoid arthritis [published online ahead of print, 2022 Jul 26]. Autoimmunity. 2022;1–12
*IL18*	Interleukin 18			
*CASP5*	Caspase 5	Ulrich C, Kneser L, et al. (2021)	34,941,677	Pyroptosis: A Common Feature of Immune Cells of Haemodialysis Patients. Toxins (Basel). 2021;13(12):839
*CASP8*	Caspase 8			
*NLRC4*	NLR Family CARD Domain Containing 4	Li Y, Yu W, et al. (2022)	35,835,791	Circ_0000181 regulates miR-667-5p/NLRC4 axis to promote pyroptosis progression in diabetic nephropathy. Sci Rep. 2022;12(1):11,994
*TNF*	Tumor Necrosis Factor	Zhu Q, Meng P, et al. (2022)	35,571,739	Luteolin induced hippocampal neuronal pyroptosis inhibition by regulation of miR-124-3p/TNF-α/TRAF6 axis in mice affected by breast-cancer-related depression. Evid Based Complement Alternat Med. 2022;2022:2,715,325
*GSDMB*	Gasdermin B	Rana N, Privitera G, et al. (2022)	35,021,065	GSDMB is increased in IBD and regulates epithelial restitution/repair independent of pyroptosis. Cell. 2022;185(2):283–298.e17
*NLRP1*	NLR Family Pyrin Domain Containing 1	Griswold AR, et al. (2019)	35,238,869	The NLRP1 inflammasome induces pyroptosis in human corneal epithelial cells. Invest Ophthalmol Vis Sci. 2022;63(3):2

### Construction of protein–protein interaction networks

The Search Tool for the Retrieval of Interacting Genes (STRING; https://string-db.org/) database was used to screen for pyroptosis-related genes in the protein–protein interaction (PPI) network. Without considering disconnected nodes, we used an interaction score > 0.4 to construct the PPI network. Subsequently, the PPI network was visualized using Cytoscape software (version 3.8.2). Pyroptosis-associated genes with degree of centrality ≥ 10 were identified as hub pyroptosis-associated genes using the cytoHubba plugin.

### Functional enrichment analysis

After identifying six pyroptosis-associated hub genes in the PPI network, we performed gene ontology (GO) analysis of molecular functions, cellular components, and biological processes, as well as Kyoto Encyclopedia of Genes and Genomes (KEGG) enrichment analysis, using the Database for Annotation, Visualization and Integrated Discover (DAVID; https://david.ncifcrf.gov/) and KEGG (https://www.kegg.jp/kegg/kegg1.html). An enrichment-adjusted *P* < 0.05 was considered statistically significant. The results of functional enrichment analysis of hub pyroptosis-related genes were visualized using the R package clusterProfiler. After categorizing glioma patients into high and low CASP4 expression groups based on their median CASP4 expression levels, Gene Set Enrichment Analysis (GSEA) was performed using the clusterProfiler package for R (v.3.6.3) to elucidate significant differences in survival, functions, and pathways between the two groups. *P* < 0.05 was considered to indicate statistical significance.

### Heatmap analysis

Heatmaps were created using TCGA expression profiles and visualized using a heatmap software package.

### Survival prognosis analysis

Based on TCGA data, Gene Expression Profiling Interactive Analysis 2 (GEPIA2; http://gepia.cancer-pku.cn/) was used to investigate the overall survival (OS) and disease-free survival (DFS) associated with the six hub pyroptosis-related genes in gliomas. Based on the median value for gene expression, we plotted Kaplan–Meier survival curves after dividing all samples into high and low CASP4 expression groups. Analyses were performed using risk ratios (95% confidence intervals) and log-rank tests. *P* < 0.05 was considered to indicate statistical significance.

### TIMER database analysis

To determine the relationship between CASP4 expression and cellular pyroptosis in gliomas, nine genes (*CASP1*, *CASP3*, *CASP5*, *GSDMD*, *AIM*, *NLRP1*, *NLRP3*, *IL-1B*, and *IL-18*) related to the classical pyroptosis pathway were analyzed for their relevance to gliomas (LGG + GBM) based on data obtained from TCGA. *P*-values were calculated for each sample, and *P* < 0.05 was considered to indicate statistical significance. The Tumor Immuno Estimation Resource (TIMER; https://cistrome.shinyapps.io/timer/), which is a comprehensive web server and includes more than 10,000 samples from multiple cancer types in TCGA, was used to determine the correlation between CASP4 expression and the abundance of classically pyroptotic cells in gliomas.

### Immune cells proportion and infiltration analysis

TIMER, which is a rich resource for the comprehensive analysis of immune infiltration in different cancer types, was used to analyze the correlation between the abundance of six immune cell types (neutrophils, CD8^+^ T cells, CD4^+^ T cells, dendritic cells, B cells, and macrophages) in infiltrates, and CASP4 expression in gliomas. We used the Cell-type Identification by Estimating Relative Subsets of RNA Transcripts (CIBERSORT) algorithm to analyze the proportion of tumor-infiltrating immune subpopulations. CIBERSORT R script (v1.03) was used to assess the relative proportions of the 22 immune cell types in glioma samples. *P*-values were calculated for each sample, and *P* < 0.05 was considered to indicate statistical significance. We performed an analysis of variance (ANOVA) to identify significant differences in the proportions of immune cells in the high and low CASP4 expression groups of glioma patients. We further evaluated the estimates of stromal and immune cells in tumor tissue using the Estimation of STromal and Immune cells in MAlignant Tumors using Expression data (ESTIMATE) algorithm. Stromal, immune, ESTIMATE scores, and tumor purity were calculated by the Estimates algorithm in the R software package. In addition, we downloaded the relevant genes and tumor immune steps relevant to the Cancer-Immunity Cycle from the Tracking Tumor Immunophenotype (TIP; http://biocc.hrbmu.edu.cn/TIP/index.jsp) database and performed correlation analysis to verify the close relationship between CASP4 and the immunotherapeutic processes for individual glioma patients.

To determine the correlations of CASP4 expression with immunosuppressant use and chemokine and chemokine receptor expression in human gliomas, we used data from the Tumor and Immune System Interaction Database (TISIDB; http://cis.hku.hk/TISIDB). Based on data obtained from TCGA, we analyzed the correlations of CASP4 expression with immunosuppressant use and chemokine and chemokine receptor expression in LGG and GBM using Spearman analysis. *P* < 0.05 was considered to indicate statistical significance.

### Glioma clinical information analysis

To further explore the relationship between CASP4 expression and the clinicopathological characteristics of glioma patients, we used patients’ age, gender, molecular subtype, pathological grade, clinical stage, IDH mutation status, 1p/19q code deletion status, and methylation status as categorical variables.

### Correlation analysis of IC50

We explored the correlation between CASP4 and glioma sensitivity to various chemotherapeutic agents using data from the Genomics of Drug Sensitivity in Cancer (GDSC; http://www.cancerrxgene.org) database and Pearson analysis.

### Cell culture

The human GBM cell line U251 was purchased from iCell Bioscience Inc. (iCell-h219, Shanghai, China) and the human astrocytes cell line HA1800 was purchased from Sciencell (No. 1800, Carlsbad, CA, USA). The cell lines were characterized by Specialized Technology Resources (STR) testing. Cell were cultured in Dulbecco’s modified Eagle’s medium (DMEM; D0822, Sigma, Saint Louis, MO, USA) supplemented with 10% fetal bovine serum (FBS; MK1128-500, MIKX Co., China) and 1% streptomycin and penicillin (100 μg/ml streptomycin and 100 U/ml penicillin) at 37 °C under 5% CO_2_.

### Transfection of cultured cells

Cultured cells were transfected with plasmid DNA using INTERFERin transfection reagent (101,000,028, Polyplus-transfection, Illkirch, France) according to the manufacturer’s instructions. After 48 h, the cells were harvested for Western blotting. The CASP4 siRNA sequence was GUGUAGAUGUAGAAGAAtt.

### Colony formation assay

U251 cells in culture medium containing 10% FBS and 1% antibiotics were seeded into 6-well plates. For siRNA treatment, 2 μg of siRNA mixed with the same volume of transfection reagent (Polyplus-transfection, Illkirch, France) was added to the cells and incubated for 6 h. The cells were then centrifuged at 800 rpm for 3 min at room temperature, washed three times with PBS, and seeded into 6-well plates (500 cells per well). All cells were cultured at 37 °C under 5% CO_2_ for 10 days and then stained with crystal violet.

### Western blot analysis

Cells were lysed with RIPA buffer, and total protein was extracted. Protein concentration was determined using a BCA protein assay kit (P0011, Beyotime, Shanghai, China) according to the manufacturer’s instructions. Subsequently, proteins were separated by 12% sodium dodecyl sulfate (SDS)-polyacrylamide gel electrophoresis (PAGE) and transferred to membranes. The corresponding proteins were detected using antibodies against CASP4 (1:1000; WL04243, Wanleibio, Shenyang, China) and GAPDH (1:2000, #2118, Cell Signaling Technology, Danvers, MA, USA). Membranes were incubated with horseradish peroxidase (HRP)-conjugated secondary antibody (1:1000, ZSGB-BIO, Beijing, China)^24^. Photographs were obtained using a fluorescence/chemiluminescence imaging system (CLINX Scientific Instruments, Shanghai, China) and analyzed using ImageJ software. Each experiment was repeated at least three times.

### Immunohistochemical staining

Tissues from three glioma patients were fixed with 10% formaldehyde and embedded in paraffin. Subsequently, the tissues were sectioned and incubated overnight at 4 °C with an anti-CASP4 antibody (1:100; WL04243, Wanleibio, Shenyang, China). After washing with PBS, sections were incubated with HRP-labeled goat anti-rabbit secondary antibody (1:200) for 30 min at room temperature. The samples were washed with PBS, incubated with DAB for 2–5 min at room temperature, then stained with hematoxylin. Micrographs of five unduplicated areas were obtained using an Olympus microscope.

### Statistical analysis

Statistical analyses were performed using ImageJ software (1.38e) and the Statistical Package for the Social Sciences (SPSS 26.0). Results were visualized using GraphPad Prism 9.2.0. Correlations were evaluated using Pearson’s or Spearman’s analyses. Student’s *t*-test was used to determine statistical significance of differences between groups. Differences between more than two groups were analyzed using one-way ANOVA. All experiments were repeated three times for validation. *P*-values < 0.05 were considered to indicate statistical significance.

### Ethical approval and consent to participate

This study was approved by the Institutional Review Board of Harbin Medical University (Approval No. KY2023-072; Harbin, China), and the study was conducted in accordance with the Declaration of Helsinki. All participants provided written informed consents to participate in this study.

## Results

### Screening and identification of pyroptosis-related genes in gliomas

**Figure 1:**
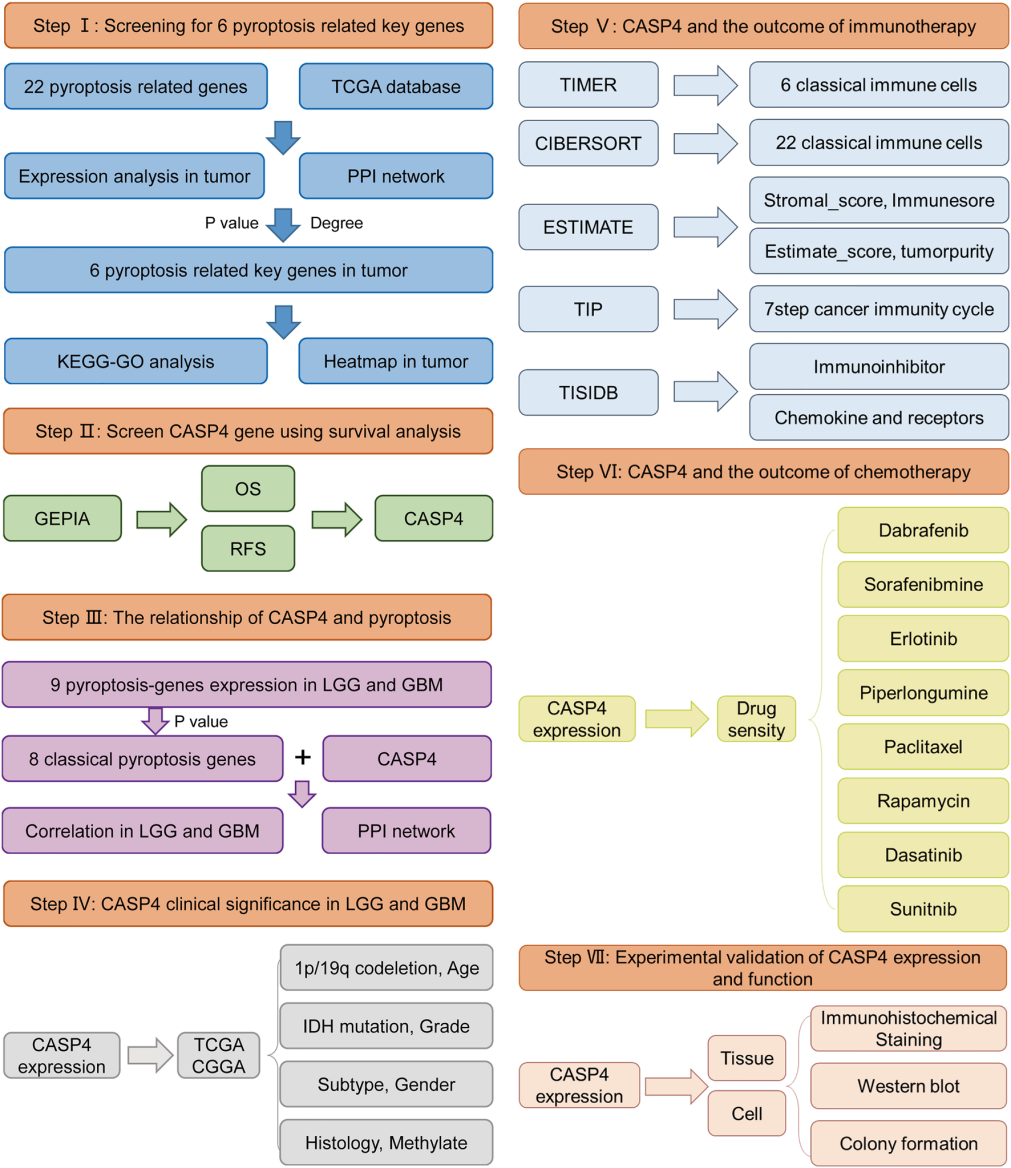
Flow diagram of study procedure.

**Figure 2:**
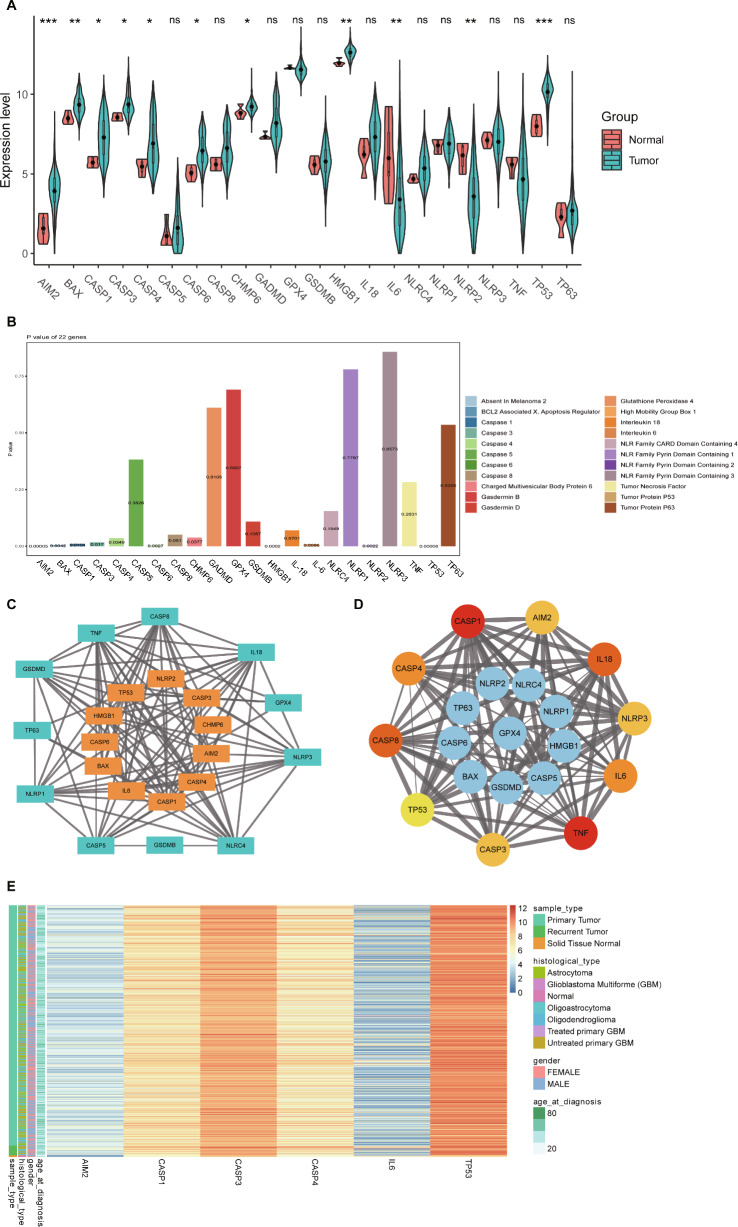
Expression and interaction network of pyroptosis-related genes in gliomas.

The process used to screen pyroptosis-related genes in gliomas is described in Fig. 1. From TCGA, we carefully screened and downloaded data on a total of 530 LGG and 167 GBM tumors and non-tumor samples for comparison. Details of the 22 possible pyroptosis-related genes that have been studied in tumor patients in recent years are shown in Table 1. A total of 11 genes associated with pyroptosis were found to be differentially expressed in glioma tumors and adjacent non-tumor tissues (Fig. 2A,B; *P* < 0.05). We used Cytoscape to visualize the relationship between the two groups of significantly differentially expressed genes on a PPI network graph (Fig. 2C, yellow: *P* < 0.05; blue: ns, *P* > 0.05). Then, to further screen for pyroptosis-related genes that play essential roles in the pathogenesis of gliomas, we performed detailed analysis of the 22 possible pyroptosis-related genes using the STRING database and sorted the pivotal genes by degree centrality using the Cytoscape plugin cytoHubba. Figure 2D shows a gene module centered on 10 pyroptosis-related genes (*AIM2*, *CASP1*, *CASP3*, *CASP4*, *CASP8*, *IL-6*, *IL-18*, *NLRP3*, *TNF*, and *TP53*). We found that six hub genes (*AIM2*, *CASP1*, *CASP3*, *CASP4*, *IL-6*, and *TP53*) were present in both sets of PPI networks (Supplementary Fig. 1). A heatmap of the expression levels of the six pivotal pyroptosis-related genes is shown in Fig. 2E. *AIM2*, *CASP1*, *CASP3*, *CASP4*, and *TP53* expression was found to be increased in glioma samples compared to non-cancer samples, whereas *IL-6* expression was significantly decreased. Furthermore, the expression levels of these genes correlated with the patient age and histological cancer type.

(**A**) Differential expression of 22 pyroptosis genes in gliomas the TCGA database. (**B**) Bar graph showing *P*-values of 22 pyroptosis genes in gliomas. (**C**) Protein–protein network of 22 pyroptosis-related in gliomas sorted by *P*-value (yellow: *P* < 0.05; blue: ns, *P* > 0.05). (**D**) Protein–protein interaction network of proteins encoded by 22 pyroptosis-related genes in gliomas sorted by degree of centrality (change from yellow to red indicates an increase in importance). (**E**) Heatmap of six hub pyroptosis-related genes (blue to red indicates low- to high-expression levels of hub pyroptosis-related genes). *P < 0.05, ^**^P < 0.01, ^***^P < 0.001, ^****^P < 0.0001 and ns, no significant difference (*P* > 0.05).

## Biological functions of the screened genes are closely related to *cancer* development

**Figure 3:**
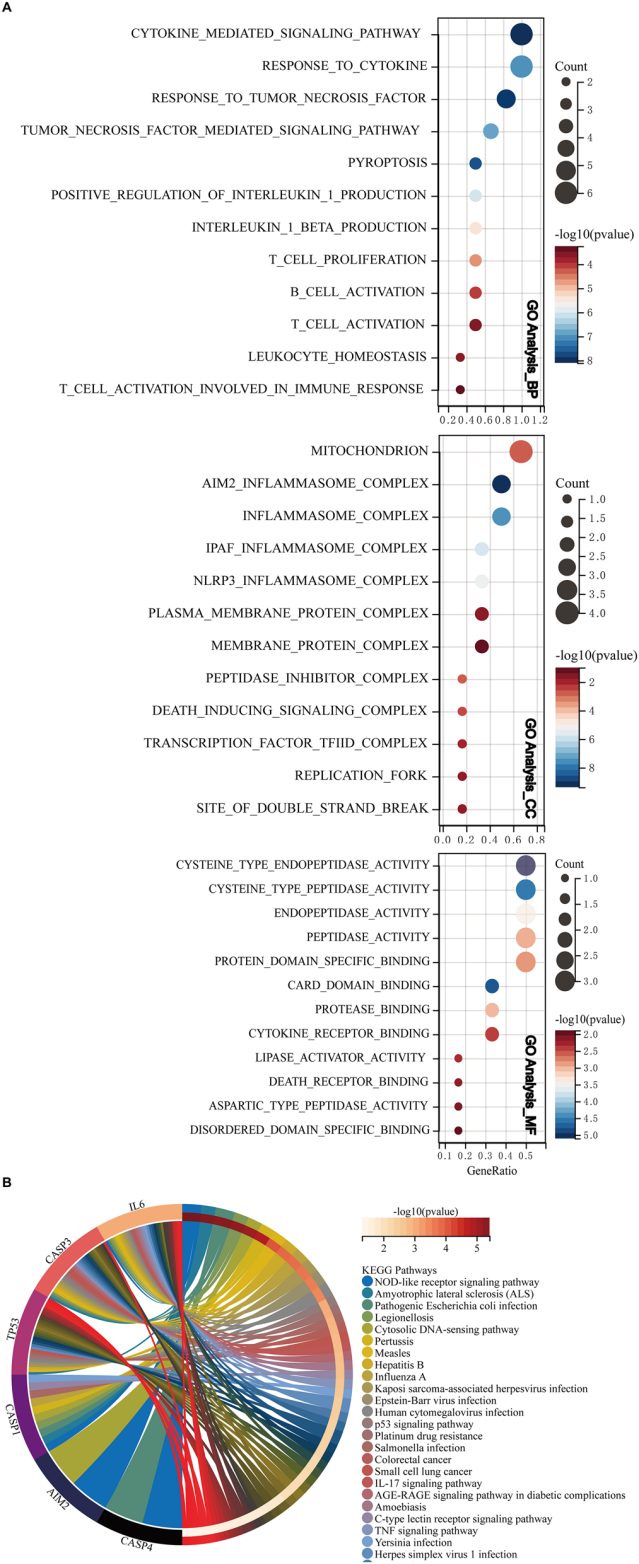
GO and KEGG enrichment analysis of six pyroptosis-related hub genes. (**A**) Biological processes (BP), Cellular components (CC), Molecular function (MF) in GO enrichment analysis. (**B**) KEGG pathway enrichment analysis.

To explore the related pathways and functions, we performed GO and KEGG enrichment analysis of the six hub genes. These genes were enriched mainly in immune, pyroptosis, and tumor-related biological processes, such as cytokine-mediated signaling pathways, responses to cytokines, responses to tumor necrosis factor, pyroptosis processes, interleukin (IL)-1β production, T cell proliferation, B cell activation, T cell activation, leukocyte homeostasis, and activation of T cells involved in the immune response (Fig. 3A). Cellular components associated with the hub genes were associated with tumor cell pyroptosis and proliferation, and cellular localizations included mitochondria, inflammasome complex, plasma membrane protein complex, membrane protein complex, peptidase inhibitor complex, death-inducing signaling complex, replication forks, and double-strand breakpoints (Fig. 3A). In addition, molecular functional analysis verified that the six hub genes were enriched mainly in cysteine-type peptidase activity, endopeptidase activity, protein domain-specific binding, protease binding, cytokine receptor binding, death receptor binding, aspartate-type peptidase activity, and disordered domain-specific binding (Fig. 3A). KEGG pathway enrichment analysis showed that the six pyroptosis-related hub genes were enriched mainly in the Nucleotide-binding Oligomerization Domain (NOD)-like receptor, cellular DNA sensing, p53, IL-17, C-type lectin receptor, and Tumor Necrosis Factor (TNF) signaling pathways, as well as platinum resistance, colorectal cancer, and small cell lung cancer (Fig. 3B). Functional analyses suggested that the six hub genes may be intimately involved in tumor cell pyroptosis, drug resistance among tumor cells, regulation of the tumor immune microenvironment, and tumor development.

### High CASP4 expression significantly correlates with the poor prognosis of glioma patients

**Figure 4:**
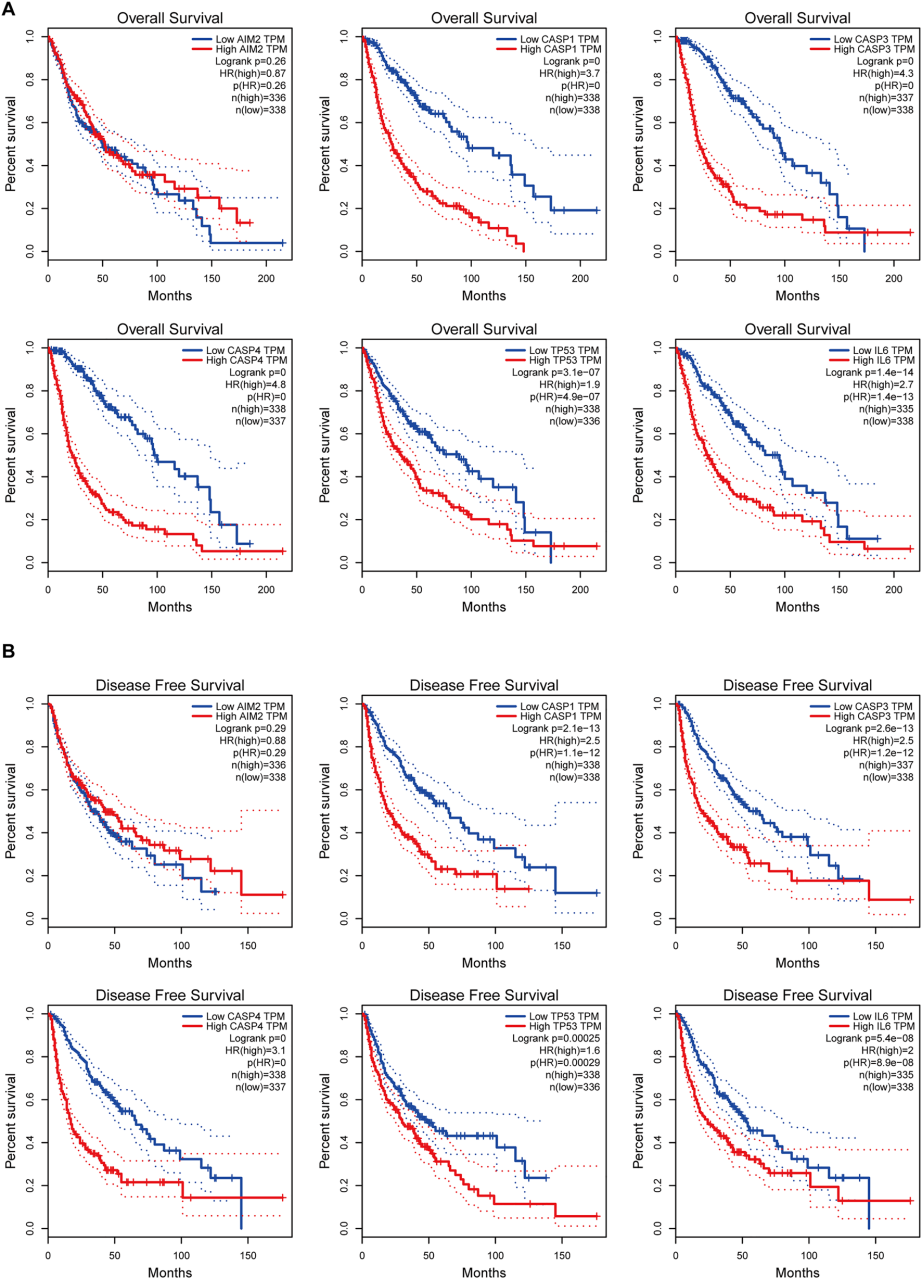
Kaplan–Meier survival curves of the six pyroptosis-related hub genes.

To assess the role of the pyroptosis-related hub genes in independent prognoses, we extracted clinical information on a cohort of glioma patients from TCGA. Based on the threshold of the median risk score, we allocated the glioma patients to high- and low-risk groups. Kaplan–Meier survival curves constructed to differentiate the OS and DFS of the glioma patients showed that CASP4 was strongly associated with OS in glioma patients (*P* < 0.0001, Fig. 4A). The correlation between the upregulation of CASP4 expression levels and poor prognosis of glioma patients was the most significant compared to changes in the other pivotal pyroptosis-related genes. Similar results were observed with corresponding DFS data (*P* < 0.0001, Fig. 4B). These findings implicate CASP4 as a prospective biomarker for gliomas. Although some reports have addressed the role of CASP4 in gliomas, to the best of our knowledge, the mechanisms underlying the role of CASP4 in immunotherapy and chemotherapy have not been fully elucidated; therefore, we selected CASP4 for further analysis.

Relationship between the expression of the six pyroptosis-related hub genes and (**A**) OS and (**B**) DFS in glioma patients allocated to high- and low-risk groups based on the threshold of the median risk score; Cutoff-High (%) = 50, Cutoff-Low (%) = 50.

### CASP4 expression is strongly associated with pyroptosis genes of patients with glioma

**Figure 5:**
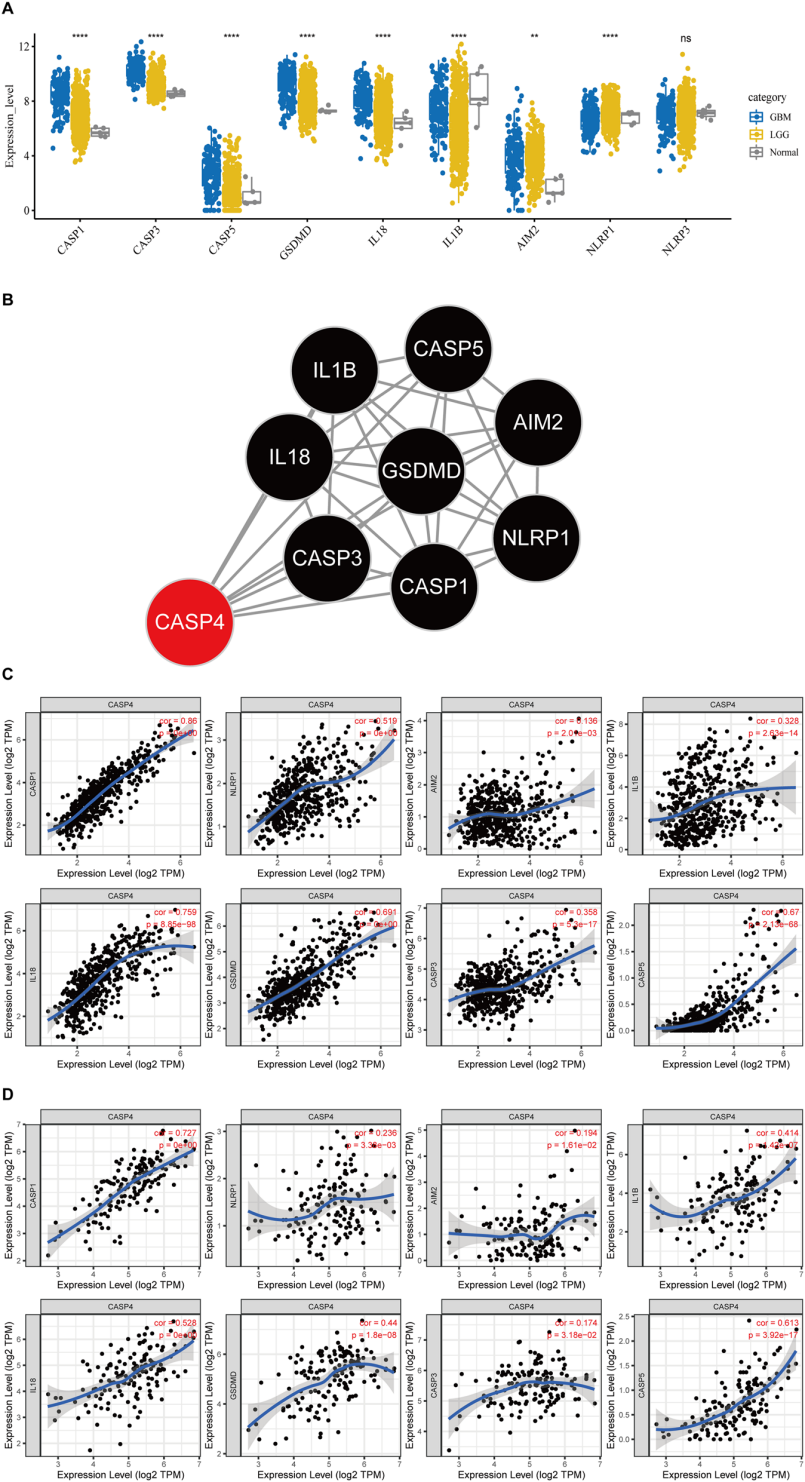
Correlation between CASP4 and classical pyroptosis-related glioma genes. (**A**) Expression of recognized pyroptosis pathway-related genes in LGG and GBM. (**B**) Protein–protein interaction analysis of CASP4 and classical pyroptosis-related genes; number of nodes = 8, number of edges = 26, average node degree = 6.5, avg. local clustering coefficient = 0.94, PPI enrichment *P*-value < 1.0e−16. (**C**) Correlation between expression of CASP4 and classical pyroptosis-related genes in LGG. (**D**) Correlation between expression of CASP4 and classical pyroptosis genes in GBM. *P < 0.05, ^**^P < 0.01, ^***^P < 0.001, ^****^P < 0.0001 and ns, no significant difference (*P* > 0.05). The significance of differences between groups of samples was evaluated by Wilcox tests (2 groups) and Kruskal–Wallis analysis (≥ 3 groups).

We first analyzed the relationship between CASP4 expression and glioma cell pyroptosis as a recently discovered form of cell death. Recent studies have shown that the caspase family triggers cellular pyroptosis through the cleavage of GSDMD protein, which releases the corresponding inflammatory factors via a pathway involving nine related proteins: CASP1, CASP3, CASP5, GSDMD, AIM2, NLRP1, NLRP3, IL-1B, and IL-18^25,26^. According to the data obtained from TCGA, eight classical pyroptosis pathway-related genes were found to be associated with the development of gliomas (LGG + GBM) (Fig. 5A). The PPI network of the proteins encoded by these eight classical pyroptosis genes and CASP4 revealed eight CASP4 node edges (Fig. 5B), indicating the importance of CASP4 in weighting the relationships among the nine pyroptosis-related hub genes. The TIMER web server was further employed to determine the correlation between CASP4 expression with the eight classical pyroptosis genes in GBM and LGG. We found that CASP4 expression was positively correlated with the eight classical pyroptosis genes in both LGG and GBM (Fig. 5C,D).

### Aberrant overexpression of CASP4 is correlated to adverse clinicopathological features of glioma

**Figure 6:**
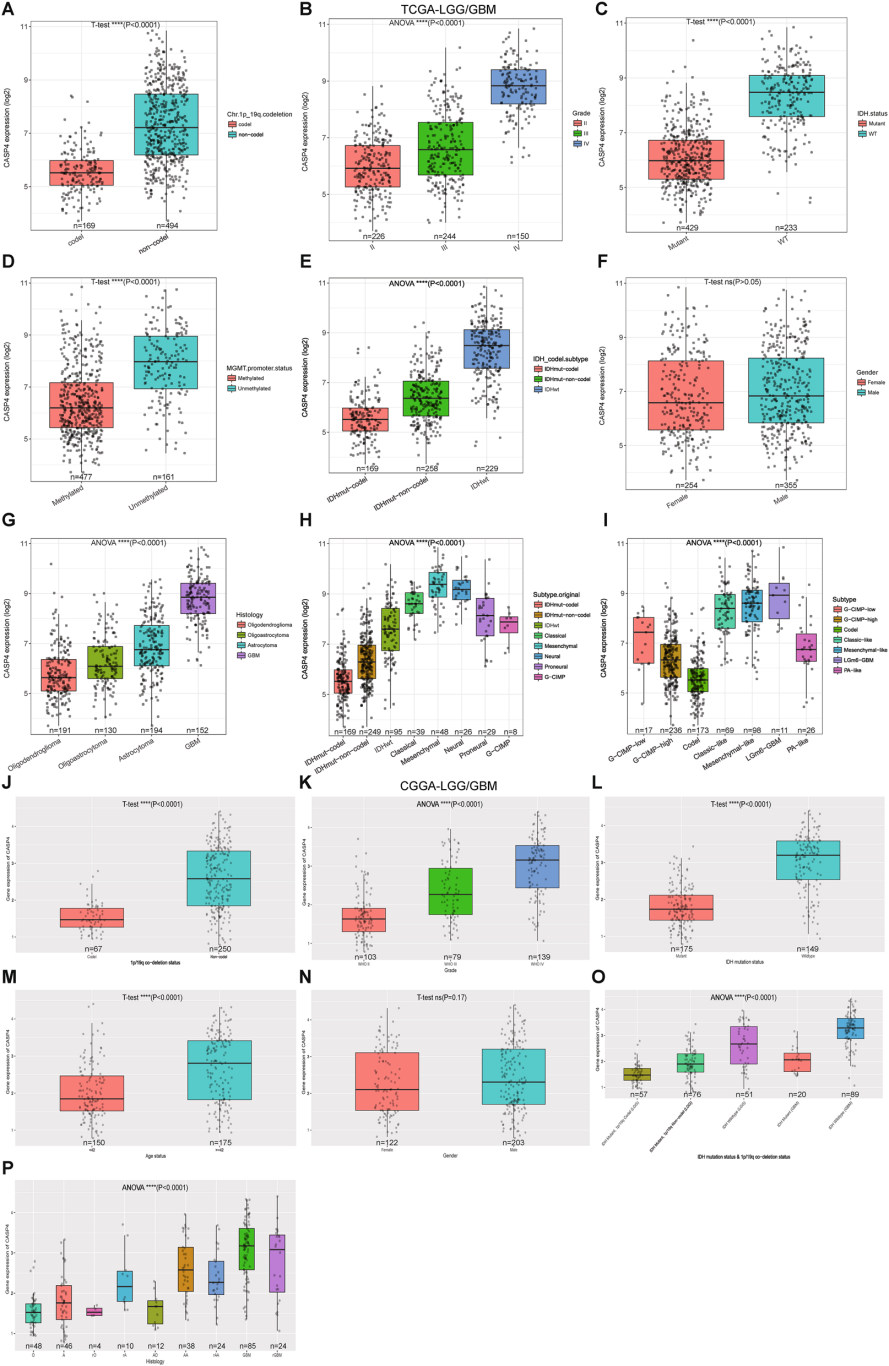
Correlation between CASP4 expression and clinical manifestations in glioma patients.

The gene expression profiles of all glioma samples and paired normal tissues showed that CASP4 was overexpressed in gliomas. In recent years, 1p/19q coding deletions, IDH mutations, and methylation of the O-6-methylguanine DNA methyltransferase (*MGMT*) gene promoter have been widely used in the diagnosis and prognostic assessment of gliomas^27^. To further explore the relationship between high CASP4 expression and the clinical presentation of glioma patients, we investigated the relationship between CASP4 expression and the characteristics of age, gender, molecular subtype, pathological grade, clinical stage, isocitrate dehydrogenase (IDH) mutation, 1p/19q codeletion, and MGMT methylation status. Graphical representation of these characteristics confirmed that CASP4 overexpression in the TCGA dataset was significantly correlated with WHO grade, IDH genotype, 1p/19q code, *MGMT* gene promoter methylation status, and age. Furthermore, we found that CASP4 expression was significantly lower in the IDH mutation group, the 1p/19q chromosome group, and *MGMT* gene promoter methylation group, and increased significantly with WHO grade and age (Fig. 6A–E). CASP4 expression also varied significantly among different histological subtypes (Fig. 6G–I), whereas there was no difference in CASP4 expression between male and female patients (Fig. 6F). These results were validated in similar evaluations of these characteristics for glioma patients in the CGGA database (Fig. 6J–P). These findings suggested that CASP4 is significantly overexpressed in gliomas and is associated with adverse clinicopathologic features.

The relationship between CASP4 expression and 1p/19q codeletion (**A**), pathological grade (**B**), isocitrate dehydrogenase (IDH) mutation status (**C**), *MGMT* gene promoter methylation (**D**), IDH mutation subtype (**E**), gender (**F**), and histological subtypes (**G-I**) in LGG and GBM of TCGA database. Correlation between CASP4 expression and 1p/19q codeletion (**J**), pathological grade (**K**), IDH mutation status (**L**), age (**M**), gender (**N**), IDH mutation, and 1p/19q codeletion status (**O**), histological subtypes (**P**) in LGG and GBM of CCGA database. *P < 0.05, ^**^P < 0.01, ^***^P < 0.001, ^****^P < 0.0001 and ns, no significant difference (*P* > 0.05).

### CASP4 expression are relevant to the development of immune cell infiltration in glioma

**Figure 7:**
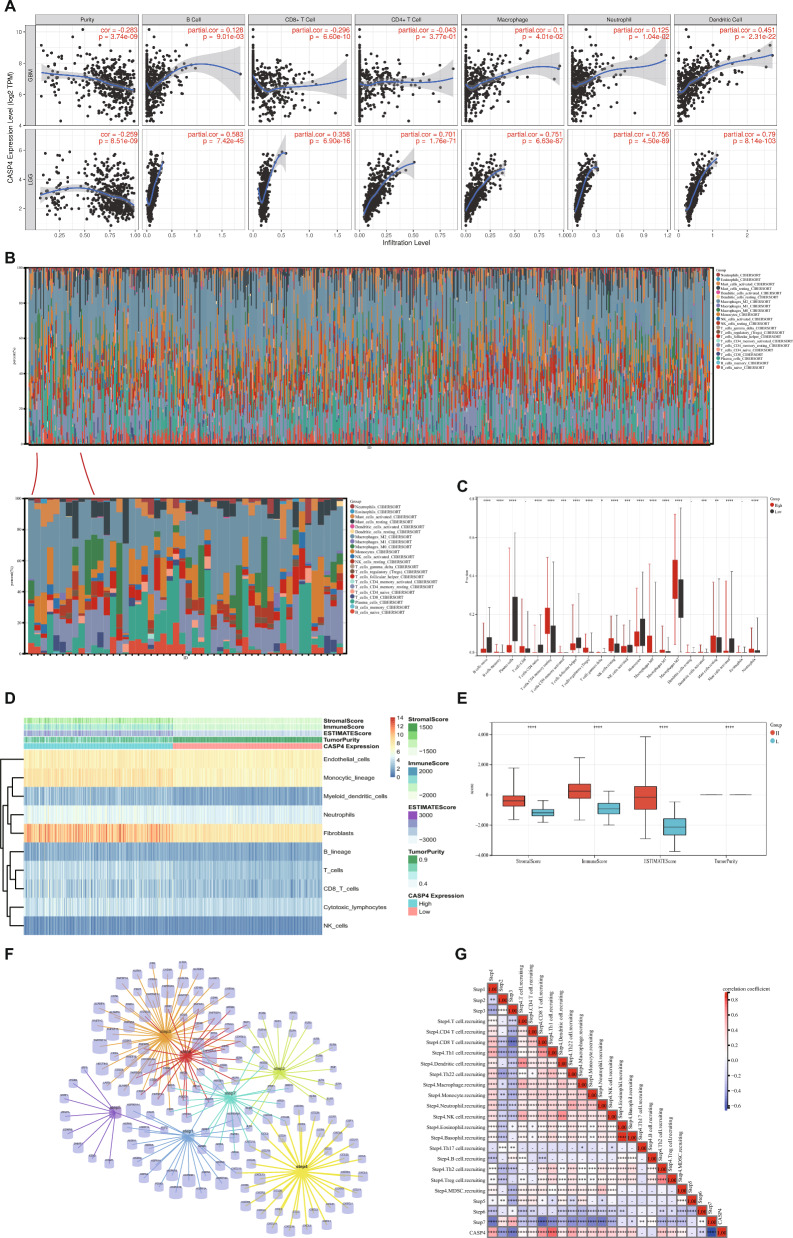
Distribution of tumor-infiltrating immune cells in glioma and their correlation with CASP4 expression. (**A**) Correlation between CASP4 and six tumor-infiltrating immune cell types in LGG and GBM. (**B**) Percentages of 22 immune cells in glioma patients determined using the CIBERSORT algorithm. (**C**) Percentage differentiation of 22 immune cells in patients with high and low CASP4 expression. (**D**) ESTIMATE algorithm results for relationship between high and low CASP4 expression with immune cells and immune scores (**E**). (**F**) Genes associated with the seven steps of antitumor immunity. (**G**) Correlation between CASP4 expression and the seven steps of anti-tumor immunity. *P < 0.05, ^**^P < 0.01, ^***^P < 0.001, ^****^P < 0.0001 and ns, no significant difference (*P* > 0.05).

As an important component of the TME, tumor-infiltrating immune cells are widely associated with the occurrence, development, and metastasis of malignant tumors^28,29^. To ascertain whether CASP4 influences immunotherapy by affecting tumor-infiltrating immune cells, we analyzed the potential association between CASP4 expression and tumor-infiltrating immune cells in multiple cancer types based on the TIMER database. We found that CASP4 expression was strongly correlated with the level of immune cell infiltration in many cancers, including colon, hepatocellular liver, squamous lung, testicular, and prostate cancers (Supplementary Fig. 2). Of particular relevance, we found that CASP4 has an important effect on tumor-infiltrating immune cells in gliomas. As shown in Fig. 7A, CASP4 expression was positively correlated with the abundance of B cells (partial correlation = 0.128, *P* = 9.01e−03), macrophages (partial correlation = 0.1, *P* = 4.01e−02), neutrophils (partial correlation = 0.125, *P* = 1.04e−02), and dendritic cells (partial correlation = 0.451, *P* = 2.31e−22), and negatively correlated with cancer purity (partial correlation = −0.283, *P* = 3.74e−09) and CD8^+^ T cells (partial correlation = −0.296, *P* = 6.60e−10) in the GBM data set. In contrast, in the LGG dataset, CASP4 expression was negatively correlated with LGG purity (r = −0.259, *P* = 8.51e−09) and positively correlated with the abundance of several immune cell types, including B cells (r = 0. 583, *P* = 7.42e−45), CD8^+^ T cells (r = 0.358, *P* = 6.90e−16), CD4^+^ T cells (r = 0.701, *P* = 1.76e−71), macrophages (r = 0.751, *P* = 6.63e−87), neutrophils (r = 0.756, *P* = 4.50e−89), and dendritic cells (r = 0.79, *P* = 8.14e−103). In general, high CASP4 expression is closely associated with increased numbers of tumor-infiltrating immune cells in gliomas. Such an association promotes poor outcomes after immunotherapy as well as worsening progression^30^.

To explore the correlation between CASP4 expression and the proportions of infiltrating immune cells, we assessed the status of 22 tumor-infiltrating immune cell types using CIBERSORT analysis. We found that the proportion of macrophages was significantly higher than that of the other immune cells (Fig. 7B). Correlation analysis showed that 18 infiltrating cell types were significantly associated with CASP4 expression (*P* < 0.01). As shown in Fig. 7C, memory B cells, resting NK cells, macrophages, memory activated CD4^+^ T cells, memory resting CD4^+^ T cells, activated dendritic cells, resting mast cells, neutrophils, and regulatory T cells (Tregs) were positively correlated with CASP4 expression, while naïve B cells, plasma cells, naive CD4^+^ T cells, follicular helper T cells, activated NK cells, monocytes, and activated mast cells were negatively correlated with CASP4 expression. We also used the ESTIMATE, immunity, and stroma scores to assess the links between CASP4 and the glioma immune microenvironment. Figures 7D,E show that high CASP4 expression was linked to higher ESTIMATE, immunity, and stromal scores, but lower glioma purity (*P* < 0.0001). In addition, we employed the TIP database to analyze the relationship between CASP4 and different aspects of tumor immunotherapy processes. Figures 7F,G show the seven-step cycle underlying the involvement of CASP4 in anticancer immunity in gliomas: Step 1, cancer cell antigen release; Step 2, cancer antigen presentation; Step 3, induction and activation; Step 4, migration of immune cells toward the tumor; Step 5, immune cell infiltration of the tumor; Step 6, recognition of the cancer cell by T cells; and Step 7, killing of the cancer cell. The data showed that high CASP4 expression is strongly correlated with the degree of migration and recruitment of immune cells to the tumor (Step 4), thus affecting the anticancer immune status and the proportion of tumor-infiltrating immune cells in glioma patients.

### CASP4 expression is closely related to the expression of immune checkpoint molecules, chemokines and chemokine receptors

**Figure 8:**
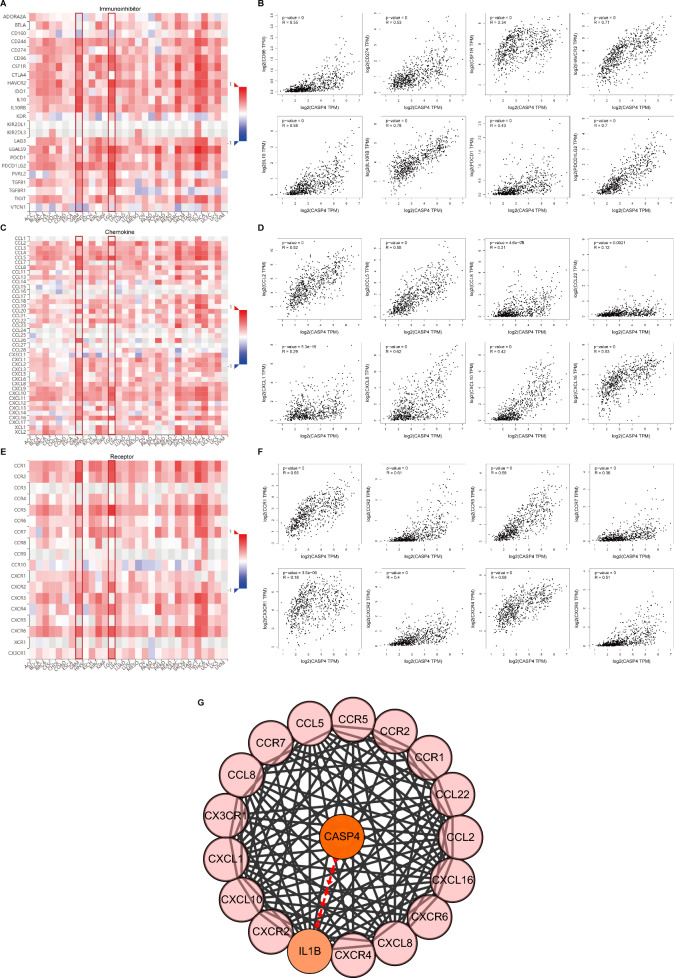
Correlations between the expression of CASP4 and immune checkpoint molecules, chemokines, and chemokine receptors. Correlations between the expression of CASP4 and immune checkpoint molecules (**A**), chemokines (**C**), and chemokine receptors (**E**) across human cancers. Scatter plots of CASP4, immune checkpoint molecules (**B**), chemokine (**D**), and chemokine receptor (**F**) expression in gliomas from TCGA dataset. (**G**) Protein–protein interaction network based on CASP4, 12 chemokines and eight chemokine receptors.

To further explore the role of CASP4 in regulating tumor immunotherapy, we investigated the association between the expression of CASP4 and immunomodulators such as immune checkpoint molecules, chemokines, and chemokine receptors using GEPIA2. In gliomas, CASP4 expression was positively correlated with the expression of major immune checkpoint inhibitor molecules, such as CD96 (r = 0.55, *P* < 0.0001), CD274 (r = 0.53, *P* < 0.0001), CSF1R (r = 0.34, *P* < 0.0001), HAVCR2 (r = 0.71, *P* < 0.0001), IL10 (r = 0.69, *P* < 0.0001), IL10RB (r = 0.78, *P* < 0.0001), PDCD1 (r = 0.43, *P* < 0.0001), and PDCD1LG2 (r = 0.7, *P* < 0.0001) (Fig. 8A,B). We also analyzed the correlation between CASP4 expression and chemokines and their receptors. CASP4 expression was positively correlated with major chemokines, such as CCL2 (r = 0.52, *P* < 0.0001), CCL5 (r = 0.55, *P* < 0.0001), CCL8 (r = 0.21, *P* < 0.0001), CCL22 (r = 0.12, *P* = 0021), CXCL1 (r = 0.29, *P* < 0.0001), CXCL8 (r = 0.62, *P* < 0.0001), CXCL10 (r = 0.42, *P* < 0.0001), and CXCL16 (r = 0.63, *P* < 0.0001) (Fig. 8C,D) and with major chemokine receptors, such as CCR1 (r = 0.65, *P* < 0.0001), CCR2 (r = 0.61, *P* < 0.0001), CCR5 (r = 0.68, *P* < 0.0001), CCR7 (r = 0.36, *P* < 0.0001), CXCR2 (r = 0.18, *P* < 0.0001), CXCR4 (r = 0.4, *P* < 0.0001), CXCR6 (r = 0.58, *P* < 0.0001), and CX3CR1 (r = 0.51, *P* < 0.0001) (Fig. 8E,F). These results implied that CASP4 has an essential role in the immunomodulation of glioma patients.

As CASP4 interactions among chemokines or chemokine receptors can affect immunotherapy and the migration of immune cells in gliomas, we used PPI network analysis to better understand the interactions. The interactions between chemokines, chemokine receptors, and CASP4 were extracted from the STRING database and then collated for visualization using the Cytoscape tool. As shown in Fig. 8G, the PPI network suggested that CASP4 interacts with the above chemokines, as well as chemokine receptors linked through IL-1β. This implied that CASP4 may be involved in immune cell migration in gliomas by regulating IL-1β protein expression. These results further support the theory that CASP4 plays an important role in glioma immunomodulation and provide new information on the immunotherapy of gliomas.

### CASP4 expression associates with clinical sensitivity to anticancer drugs

**Figure 9:**
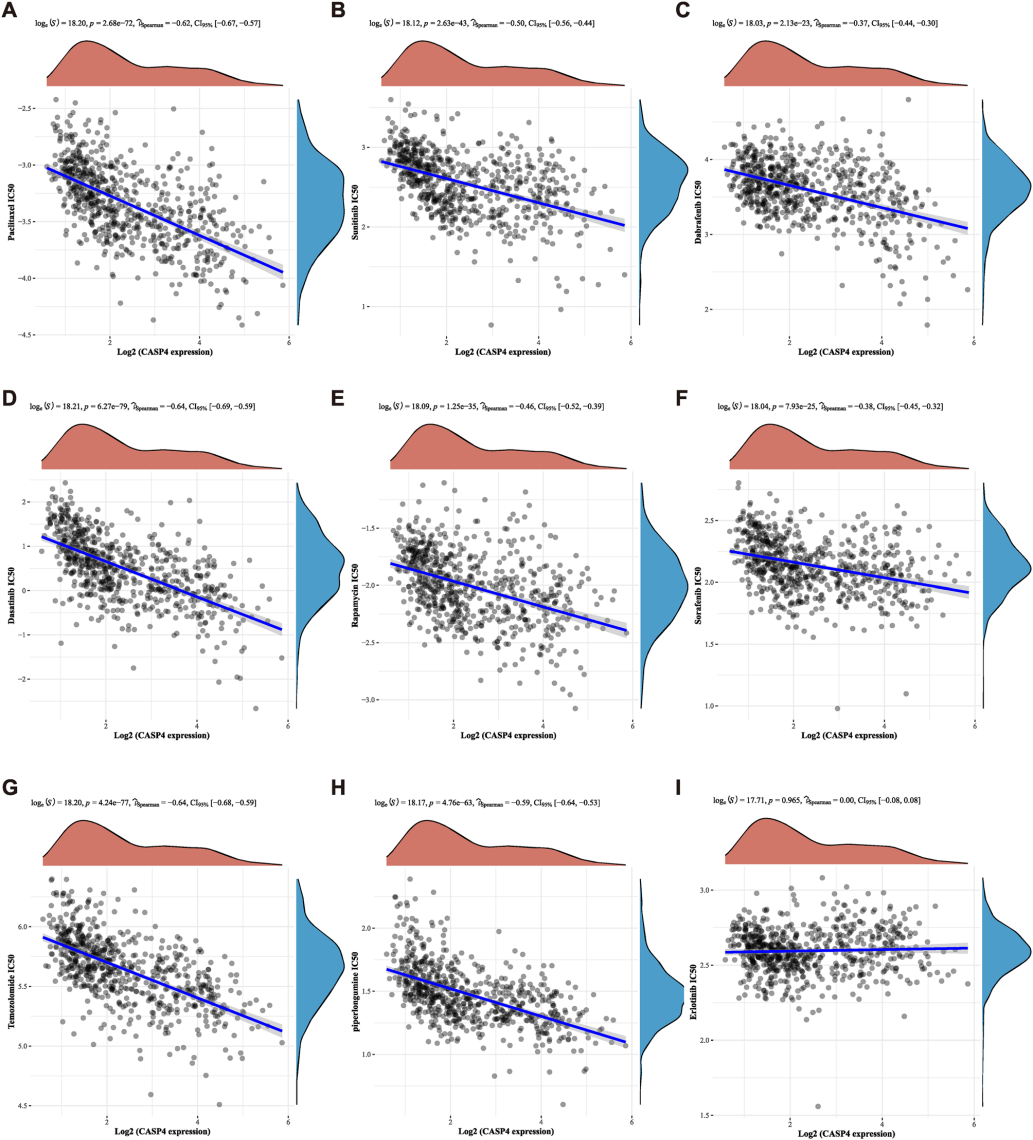
Relationship between CASP4 expression and drug sensitivity. Spearman’s correlation analysis of paclitaxel (**A**), sunitinib (**B**), dabrafenib (**C**), dasatinib (**D**), rapamycin (**E**), sorafenib (**F**), temozolomide (**G**), piperlongumine (**H**), and erlotinib (**I**) IC_50_ scores and CASP4 gene expression.

Given the potential role of CASP4 in driving glioma progression, we analyzed the relationship between CASP4 expression and glioma chemotherapy treatment using the GDSC database. CASP4 expression was negatively correlated with drug sensitivity for most chemotherapeutic agents, including paclitaxel (Pearson correlation ρ = − 0.62, *P* < 0.0001), sunitinib (Pearson correlation ρ = − 0.50,* P* < 0.0001), dabrafenib (Pearson correlation ρ = − 0.37, *P* < 0.0001), dasatinib (Pearson correlation ρ = − 0.64, *P* < 0.0001), rapamycin (Pearson correlation ρ = − 0.46, *P* < 0.0001), sorafenib (Pearson correlation ρ = − 0.38, *P* < 0.0001), temozolomide (Pearson correlation ρ = − 0.64, *P* < 0.0001), and piperlongumine (Pearson correlation ρ = − 0.59, *P* < 0.0001) (Fig. 9A–I). This indicated that inhibiting CASP4 expression increases the sensitivity of cancer cells to most chemotherapeutic agents. This may provide a new direction for drug selection and the development of synergistic agents for the chemotherapeutic management of gliomas.

### Validation of CASP4 expression and function in cells and tissues of glioma

**Figure 10:**
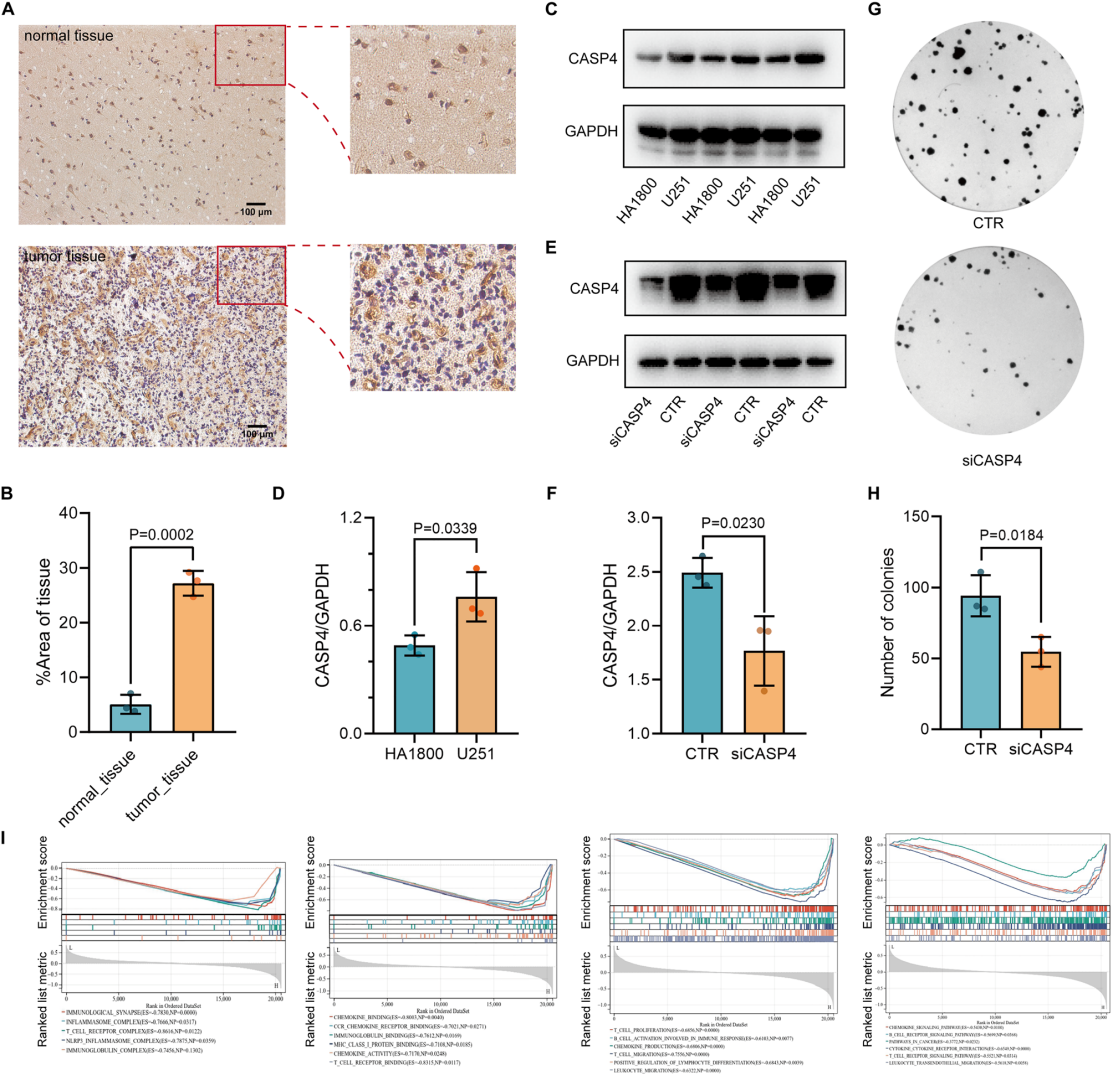
Validation of CASP4 expression and function in glioma. (**A**) CASP4 expression in normal tissue and glioma tissue. Scale bar, 100 μm. (**B**) Quantification of CASP4 protein levels in normal tissue and tumor tissue, Data represent the mean ± SD, n = 3. (**C**) CASP4 expression in astrocytes HA1800 and glioma cells U251 detected using Western blot analysis; GAPDH was used as an internal control. (**D**) Quantification of CASP4 protein levels in HA1800 and U251 cell lines. Data represent the mean ± SD, n = 3. (**E**) Efficiency of CASP4 inhibition mediated by siRNA detected by Western blot; GAPDH was used as an internal control. (**F**) Quantification of CASP4 protein inhibition mediated by siRNA. Data represent the mean ± SD, n = 3. (**G**) CASP4 inhibition significantly inhibited U251 cell migration as determined by colony formation assay. (**H**) Quantification of the number of colonies. Data represent the mean ± SD, n = 3. (**I**) Important signaling pathways associated with CASP4 according to GSEA.

To confirm the expression of CASP4 at the cancer cell and tissue level, we collected glioma cells and histopathological sections from patients. In accordance with the results from TCGA data, our analysis showed that CASP4 expression was elevated in glioma cells compared with normal astrocytes, and CASP4 was highly expressed in gliomas compared with normal paracancerous tissues (Fig. 10A–D). To further validate the effect of CASP4 on glioma cells, we inhibited CASP4 expression in U251 glioma cells and verified the inhibition efficiency by Western blot (Fig. 10E,F). Colony formation assays showed that inhibition of CASP4 expression significantly suppressed cell proliferation (Fig. 10G,H). Finally, GSEA confirmed the involvement CASP4 upregulation in pathways related to tumor cellular pyroptosis and immunity (Fig. 10I). Therefore, our comprehensive analyses indicated that CASP4 is closely associated with cellular pyroptosis, immunotherapy, and drug treatment of gliomas, suggesting the potential of CASP4 as a novel therapeutic target in gliomas.

## Discussion

Gliomas are the most common malignant brain tumors, and despite the evolution of traditional treatments such as radiotherapy chemotherapy and immunotherapy, their prognosis has changed little over the past few decades. The invasiveness, recurrence, and drug resistance of gliomas are the main reasons for the poor prognosis of patients^31,32^. Therefore, exploring the mechanisms and useful targets associated with improved immunotherapy and chemotherapy of glioma patients is the focus of contemporary neurological research. Pyroptosis is an emerging mode of cell death that may have a dual role in gliomas^33^. Risk models composed of genes or long non-coding RNAs related to pyroptosis have shown good predictive and diagnostic value in gliomas, although the exploration of key genes and therapeutic targets of pyroptosis has not been comprehensive, and there is a lack experimental evidence^34,35^. In our study, we targeted the key gene *CASP4* in a differential analysis of pyroptosis-related genes in gliomas and their relationship to clinical prognosis. Relying on comprehensive bioinformatics analyses, we demonstrated that differences in the degree of CASP4 expression are strongly associated with the prognosis of patients with gliomas. CASP4 expression may serve as an independent prognostic marker, as it correlates with a variety of clinical features, particularly the pathological stage and status of the tumor. In relation to the poor outcomes of immunotherapy and drug therapy in glioma patients, we found that glioma CASP4 expression correlated with the degree of infiltration of multiple immune cell types and sensitivity to chemotherapeutic agents. These findings suggest that CASP4 expression affects the efficacy of immunotherapy and chemotherapy of gliomas and implicate CASP4 is a promising therapeutic target.

Based on recent data from TCGA, we analyzed 22 differentially expressed pyroptosis-related genes in glioma patients, and then combined the results of two protein interactions analyses to identify six key pyroptosis-related genes in gliomas. To gain a more in-depth and systematic understanding of the biological roles of these genes, we performed GO and KEGG analyses. GO analysis revealed that the functions of the six pivotal pyroptosis genes are enriched mainly in tumor immunity, cellular pyroptosis, and other tumor-related biological processes and are closely related to glioma development, including cytokine-mediated signaling pathways that can directly affect the progression of malignant gliomas. Cytokines maintain the precise balance between glioma cells and the TME, thus contributing to the synergistic immunological and pharmacological treatment of gliomas^36^. Cellular localization analysis revealed that some of the six pivotal pyroptosis genes were localized in sites such mitochondria and the inflammasome complex. In the clinic, both mitochondrial transplantation and anti-inflammatory complexes are highly promising therapeutic modalities for gliomas, which may indicate an important function of pyroptosis genes in glioma therapy^37,38^. KEGG pathway enrichment analysis showed that the six pivotal pyroptosis genes were enriched mainly in the NOD-like receptor and p53 signaling pathways and platinum resistance, the roles of which in gliomas have been widely confirmed. The NOD-like receptor signaling pathway is central to the pathogenesis of many cancers and neurodegenerative diseases, and NOD-like receptor signaling contributes to the angiogenesis of gliomas, with NLRP3 being an important component, which promotes the growth and invasion of gliomas through IL-1β/NF-κB p65 signaling^39,40^. It has been suggested that the p14ARF-MDM2-p53 pathway is one of the major pathways in the molecular genetic pathogenesis of gliomas, in which p14ARF and MDM2 proteins are involved in glioma development by regulating the ubiquitination-mediated degradation of p53. As one of the most important oncogenic proteins, mutations in p53 can lead to the aberrant proliferation of tumor cells and promote the deterioration of cerebral glioma^41^. All of these gene function analyses indicate that the six key genes are involved in tumor cell pyroptosis, tumor immune microenvironment regulation, and tumor development through multiple pathways. Using the online database GEPIA2, we found that the correlation between CASP4 expression levels and poor glioma prognosis (OS and DFS) was the most significant, suggesting that CASP4 plays a critical role in the prognosis of glioma treatment. To the best of our knowledge, the current literature on the role of CASP4 in glioma immunotherapy and chemotherapy is superficial and limited in scope; therefore, we chose to conduct an in-depth and comprehensive analysis of the role of CASP4 in the treatment of glioma.

CASP4 plays a key role in a variety of diseases such as spinal cord injury and Alzheimer’s disease-related synaptic and behavioral deficits, and its role in tumors seems to be two-fold^13,42–44^. We explored the relationship between CASP4 and tumor behavior, tumor subtype, and other clinical features of gliomas. By exploring TCGA and CGGA, we found that CASP4 was significantly associated with WHO class, *IDH* genotype, 1p/19q coding, *MGMT* gene promoter methylation, and age. 1p/19q gene deletion, *IDH* gene mutation and *MGMT* gene promoter methylation have been widely studied and identified as diagnostic and predictive markers for glioma typing and tumor progression^45^. CASP4 may guide the development of different types of gliomas, and it is expected to make an important contribution to the prediction of glioma typing and the development of individualized treatments. One of the main reasons for the individual variability in the effects of immunotherapy on the prognosis of patients with glioma may be differences in the proportions of immune cells in the tumor infiltrate. Therefore, we used various immunoassay databases, such as TIMER, CIBERSORT, ESTIMATE, and TIP, to determine the type of immune cell infiltration that is most closely associated with CASP4. Although the TIMER immunoassay database showed that CASP4 is closely associated with dendritic cells in glioma patients, the percentage of dendritic cells in glioma immune cells, as revealed by the CIBERSORT algorithm, is very small. Therefore, dendritic cells, although closely associated with CASP4 expression, may not be the main reason why CASP4 affects immunotherapy in gliomas. Using the CIBERSORT algorithm, we found that M2 type macrophages were not only present at a high percentage, but also exhibited greater infiltration of gliomas expressing high levels of CASP4. Macrophages can promote tumor development by regulating tumor cell metabolism and angiogenesis^46^. As one of the major regulatory components in the TME, the role of tumor-associated macrophages (TAMs) in gliomas has attracted much attention because they are significantly associated with glioma progression, grading, and prognosis. Pleiotrophin secretion by TAM promotes signaling in glioblastoma stem cells and tumor growth^47^. TAMs can be functionally subtyped according to their polarization status (i.e., M1 and M2), and several studies have demonstrated a pro-tumorigenic role for M2 TAMs in gliomas^48^. Preventing TAM polarization to the M2 subtype has been reported to block glioma progression and tumor growth^49^. Our study showed that CASP4 expression followed the same trend as macrophage M2 infiltration, suggesting that CASP4 may have an impact on the associated immune cell infiltration, thereby affecting the susceptibility of glioma patients to immunotherapy. Furthermore, previous studies have shown that the expression of some combinations associated with immune steps is also a prognostic predictor for glioblastoma, e.g., the combined expression of CD276, GATA3, and LGALS3, as well as the expression of PD-L1, PD-L2, and PD-1 predicted the prognosis of glioma patients^50,51^. Based on the critical role of immune scores and steps in glioma treatment, our study identified a strong correlation between CASP4 expression and ESTIMATE scores and immune steps in glioma patients. In ESTIMATE and TIP immunoassay analyses, CASP4 expression significantly affected ESTIMATE scores and anticancer immune steps, including cancer cell antigen release, cancer antigen presentation, induction and activation, immune cell transfer to the tumor, immune cell infiltration of the tumor, T cell recognition of the cancer cells, and killing of the cancer cells^52^. Immunologic drugs affect the course of immunotherapy by targeting these seven steps. Investigating the role of CASP4 in influencing these steps has the potential to improve the efficacy of immunotherapy for glioma, making CASP4 an important predictor of accurate glioma treatment outcomes.

Considering the important role of chemokines in immune cell migration, we further explored whether the role of CASP4 in the immune microenvironment of gliomas is related to this type of regulatory factors. As an immune checkpoint receptor, CD96 has been extensively demonstrated to have biological functions in T cells and natural killer cells^53^, playing an essential role in immunotherapy and the poor prognosis of many cancers, including hepatocellular carcinomas and gliomas^54,55^. CSF1R regulates macrophage activation, proliferation, and phagocytosis mainly in the tumor immune microenvironment^56^. CSF1R inhibition alters macrophage polarization, thereby blocking glioma progression, and has been identified as a highly promising therapeutic method^49^. Similarly, CCL2/CCR2 affects the recruitment of TAMs, which in turn influences prognosis^57^. CCL5/CCR5, on the other hand, affects the efficacy of tumor therapy mainly by promoting inflammatory responses as well as inducing the adherence and migration of different T cell subsets involved in the immune response^58^. In this study, using TISIDB and correlation analysis, we identified a close correlation between CASP4 and the above-mentioned regulators, in addition to positive correlations between CASP4 and HAVCR2, IL10, CXCL8, CCR5, CCR7, CXCR2, and other immune cell-associated regulators. To further illustrate how CASP4 affects many of the above factors and thus, plays a key role in gliomas, we performed functional interplay analysis and identified the key molecule IL-1β using PPI network analysis. IL-1β is an important downstream factor that exerts the pro-inflammatory effects of pyroptosis^18^. Numerous studies have demonstrated the crucial role of IL-1β in T cell polarization, acute inflammation, and adaptive anti-tumor responses; whereas, in chronic inflammatory processes, the IL-1β produced by tumor-infiltrating macrophages may play a supportive role in tumor development^59,60^. The above results suggest that CASP4 influences the activation, proliferation, and infiltration of immune cells via the interaction of IL-1β with various immunomodulatory factors, chemokines, and their receptors, to affect the efficacy of immunotherapy for gliomas.

Drug therapy is the conventional approach to tumor treatment, and decreased drug sensitivity is an important factor that affects the progression of most tumors. Due to the limitations of radiotherapy for the treatment of neurological tumors, many studies have focused on improving the sensitivity of glioma cells to chemotherapeutic agents such as paclitaxel^61^. For this reason, we investigated the correlation between CASP4 and chemotherapeutic drug sensitivity with the aim of providing possible targets for improving the efficacy of chemotherapy in glioma patients. Paclitaxel is regarded as a classical antitumor drug because of its unique mechanism of action, although the incidence of tumor resistance is becoming increasingly widespread and is found in a diversity of cancers, such as triple-negative breast cancer and lung cancer^62,63^. The issue of decreased sensitivity is also inevitable in the clinical application of sunitinib. Jiang et al. suggested that elevated SNRPA1 predicts the sensitivity of clear cell renal cell carcinoma to sunitinib^64^. Based on our study, we propose that the important pyroptosis gene *CASP4* can be used to predict the sensitivity of glioma cells to paclitaxel and sunitinib. In addition, we found sensitivities to several other well-known antitumor drugs, such as dabrafenib, dasatinib, rapamycin, that are also closely related to CASP4 expression. Further studies on the specific mechanisms of CASP4 expression and drug resistance could provide new ideas for future chemotherapy development.

Finally, we verified the aberrant expression of CASP4 by in vitro analysis of cells and histological samples from glioma patients. Furthermore, we showed that CASP4 expression affected glioma cell proliferation. Finally, the results of GSEA indicated that CASP4 is involved in the glioma-associated NLRP3-inflammation complex as well as immune signaling pathways such as T cell activation and proliferation and chemokine activation. These pathways affect tumor immunotherapy, drug therapy, and patient prognosis in gliomas, and CASP4 has demonstrated its potential as a prognostic predictor and therapeutic target.

## Conclusion

CASP4 is a predictive therapeutic target that is closely related to immunotherapy and resistance to chemotherapeutic agents in gliomas. Our comprehensive and detailed analysis suggests that CASP4 may improve the prognosis of glioma patients mainly by affecting the immune microenvironment of the tumor, and the reduction of its expression level is an important guideline for improving the efficacy of immunotherapy and chemotherapy in glioma patients with different phenotypes and at different stages.

### Supplementary Information

Supplementary Information 1.

Supplementary Figure 1.

Supplementary Figure 2.

Supplementary Information 2.

Supplementary Information 3.

## Data Availability

The data that support the findings of this study are available in TCGA at https://portal.gdc.cancer.gov and http://www.cgga.org.cn/index.jsp.
