# Supplementary figures and images for: Caspase-4 in glioma indicates deterioration and unfavorable prognosis by affecting tumor cell proliferation and immune cell recruitment
Source: Sci Rep. 2024 Jul 29;14:17443. doi: 10.1038/s41598-024-65018-z (PMC11286837; doi:10.1038/s41598-024-65018-z)

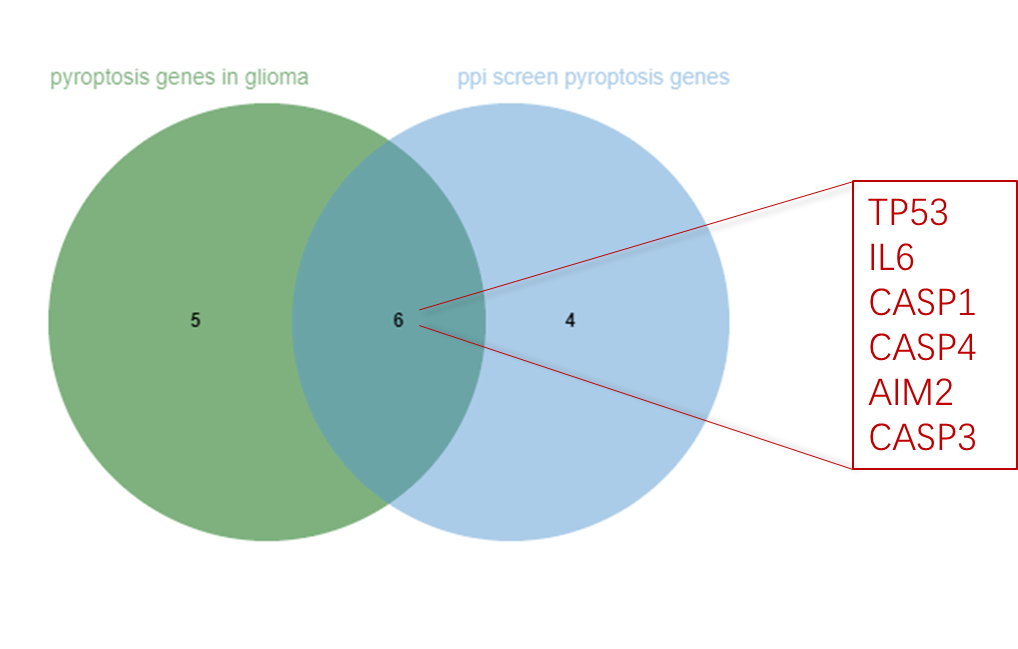

Supplement: Supplementary file 2 — Supplementary Figure 1. [file 41598_2024_65018_MOESM2_ESM.tif]
